# Toddlers’ Fine Motor Milestone Achievement Is Associated with Early Touchscreen Scrolling

**DOI:** 10.3389/fpsyg.2016.01108

**Published:** 2016-08-02

**Authors:** Rachael Bedford, Irati R. Saez de Urabain, Celeste H. M. Cheung, Annette Karmiloff-Smith, Tim J. Smith

**Affiliations:** ^1^Biostatistics Department, Institute of Psychiatry, Psychology and Neuroscience, King’s College LondonLondon, UK; ^2^Centre for Brain and Cognitive Development, Birkbeck, University of LondonLondon, UK; ^3^Psychological Sciences, Birkbeck, University of LondonLondon, UK

**Keywords:** touchscreen, tablet, infant, toddler, developmental milestones, fine motor

## Abstract

Touchscreen technologies provide an intuitive and attractive source of sensory/cognitive stimulation for young children. Despite fears that usage may have a negative impact on toddlers’ cognitive development, empirical evidence is lacking. The current study presents results from the UK Toddler Attentional Behaviours and LEarning with Touchscreens (TABLET) project, examining the association between toddlers’ touchscreen use and the attainment of developmental milestones. Data were gathered in an online survey of 715 parents of 6- to 36-month-olds to address two research questions: (1) How does touchscreen use change from 6 to 36 months? (2) In toddlers (19–36 months, i.e., above the median age, *n* = 366), how does retrospectively reported age of first touchscreen usage relate to gross motor (i.e., walking), fine motor (i.e., stacking blocks), and language (i.e., producing two-word utterances) milestones? In our sample, the proportion of children using touchscreens, as well as the average daily usage time, increased with age (youngest quartile, 6–11 months: 51.22% users, 8.53 min per day; oldest quartile, 26–36 months: 92.05% users, average use of 43.95 min per day). In toddlers, aged 19–36 months, age of first touchscreen use was significantly associated with fine motor (stacking blocks), *p* = 0.03, after controlling for covariates age, sex, mother’s education (a proxy for socioeconomic status) as well as age of early fine motor milestone achievement (pincer grip). This effect was only present for active scrolling of the touchscreen *p* = 0.04, not for video watching. No significant relationships were found between touchscreen use and either gross motor or language milestones. Touchscreen use increases rapidly over the first 3 years of life. In the current study, we find no evidence to support a negative association between the age of first touchscreen usage and developmental milestones. Indeed, earlier touchscreen use, specifically scrolling of the screen, was associated with earlier fine motor achievement. Future longitudinal studies are required to elucidate the temporal order and mechanisms of this association, and to examine the impact of touchscreen use on other, more fine-grained, measures of behavioral, cognitive, and neural development.

## Introduction

Family ownership of touchscreen devices such as tablets and mobile phones has increased in the UK from 7% in 2011 to 71% in 2014 (Ofcom, 2014). Touchscreens provide an intuitive and attractive source of sensory and cognitive stimulation for young children (Cristia and Seidl, 2015), and the impact of such devices on children’s development is a pressing question of concern to parents, scientists, and policy makers. Several prominent voices have sparked fears in the popular press about the negative impact of such technology (e.g., Carr, 2010; Sigman, 2012; Greenfield, 2015), with one of the most prominent parent-advisory agencies, the American Academy of Pediatrics (AAPs), advising zero screen time before the age of 2 years (Brown, 2011; Strasburger and Hogan, 2013; although the AAP are currently in the process of revising the guidelines). This guideline has been adopted by other government agencies around the world, including in the UK (Public Health England, 2013), Canada (Lipnowski et al., 2012), and Australia (Australian Department of Health and Ageing, 2014). However, empirical evidence relating early touchscreen use in toddlerhood to delays in cognitive development is currently lacking. Here, we examine the relationship between touchscreen use and the achievement of developmental milestones, using data from a large UK survey: the Toddler Attentional Behaviours and LEarning with Touchscreens (TABLET) project.

In line with Ofcom’s (2014) report pointing to a sharp increase in the prevalence of household touchscreen devices (Ofcom, 2014), recent studies suggest that the majority of infants and toddlers have experienced some degree of exposure to touchscreens (Ahearne et al., 2015; Cristia and Seidl, 2015; Kabali et al., 2015). In a sample of 450 babies from a French babylab (Cristia and Seidl, 2015), 58% of 5- to -24-month-old infants had used a touchscreen. This is in comparison to an earlier report that 33% of American infants (birth to 2 years of age) had used a touchscreen (Rideout, 2013). Frequency of use across these two samples was similar, with just over 20% of infants and toddlers experiencing daily use of the touchscreen. A more recent study using a low income ethnic minority American sample reported much higher frequencies with 75% of children using a touchscreen device daily by 2 years of age (Kabali et al., 2015). This percentage was mirrored (71%) in a diverse socioeconomic status (SES) hospital-based sample of 12–36 month-olds from Northern Ireland (Ahearne et al., 2015).

The type of touchscreen usage also changes with age (Cristia and Seidl, 2015). Parents report that around 75% of toddlers use touchscreens to look at photos or to watch videos, with about 50% actively playing baby-friendly apps. The increase in active use may be due to developing fine-motor skills (e.g., precise finger control), increasing executive function required to understand the touchscreen interface, as well as the developing need for more structured sensory reward and stimulation. Motor developments are demonstrated in the gestures toddlers use to interact with a screen such as banging the screen (16%), tapping (71%), dragging (41%), swiping (20%) and pinching (10%), with all gestures increasing with age (with the exception of a decrease for the non-deliberate banging gesture; Cristia and Seidl, 2015) together with the child’s need for parental assistance decreasing from 71.8% at 2 years to 57.1% at 4 years of age (Kabali et al., 2015). These usage statistics suggest that the frequency, type, and complexity of touchscreen use develops along with general cognitive development. However, it is not currently known if and how the two developmental strands interact.

How might toddler touchscreen use influence cognitive development? Given the relatively recent introduction of touchscreen devices into the developmental environment of children, there is currently no research directly assessing the impact of touchscreen use on early cognitive development. Empirical evidence from more established media, namely TV viewing and videogames, suggests that TV screen time is associated with delayed language (Zimmerman et al., 2007), poorer health (Strasburger et al., 2012), and attentional problems (Christakis et al., 2004). However, several of these effects have been shown to be moderated by factors such as parenting style (Linebarger et al., 2014), type of content (Linebarger and Walker, 2005) or coviewing with a parent (Mendelsohn et al., 2010) and may disappear when confounds such as SES are factored in Schmidt et al. (2009). Evidence for the impact of actively playing videogames is similarly mixed. Increased gaming in older children is related to greater parent/teacher-reported attentional problems (Swing et al., 2010), as well as memory and sleep problems (Dworak et al., 2007) but has also been shown to increase performance on cognitive tests including enhanced visual processing, attentional, and motor control in adults (Green and Bavelier, 2008).

Touchscreens combine the interactivity of a videogame with the non-interactive entertainment of television, but less is known about the direct impact of touchscreen use on behavior than these two established forms of media. One recent study in adults showed that touchscreen phone users (compared to non-users) have greater activation of the somatosensory cortex in response to a mechanical touch to the thumb, index finger, and middle finger (Gindrat et al., 2015). However, increased time spent with a touchscreen may also have detrimental effects on physical activity and language. The *displacement hypothesis* (Strasburger et al., 2012) states that the time a child spends engaged with a screen limits the time they have to do other activities, leading to reduced physical activity (Sisson et al., 2010; although see Taveras et al., 2007) and reduced face-to-face communication (Huttenlocher, 1998; Hart and Risley, 2003; Rowe, 2012; Sosa, 2016).

To our knowledge, no studies have directly assessed the impact of touchscreen use on toddlers’ developmental milestones. Critically, touchscreen devices can be easily used by children with relatively immature cognitive and behavioral abilities, at an age when neural development and plasticity is high (e.g., Huttenlocher, 2002). As with TV and videogames, the impact of touchscreen use on development is likely to be mixed depending on the type of use (Hirsh-Pasek et al., 2015). In the current paper we examine two main research questions: (1) How does touchscreen usage change with age across our full sample of 6- to 36-month-olds? (2) In toddlers (aged 19–36 months), is retrospectively reported age of first touchscreen usage associated with developmental milestones: gross motor (i.e., walking), fine motor (i.e., stacking blocks), and language (i.e., producing two-word utterances)?

## Materials and Methods

### Participants

In total, 715 UK-based parents of 6- to 36-month-old children completed an online questionnaire asking questions about demographic information, their child’s media usage and retrospectively reported developmental milestones. The questionnaire was administered between June 2015 and March 2016. The final sample size used in each analysis varied due to missing data for certain questionnaire elements (see **Table 1**). Parents were recruited via the Birkbeck Babylab database, Goldsmiths’ Babylab database and study advertisements from various news agencies, magazines and agencies including National Childbirth Trust (NCT). The study was approved by the Birkbeck Psychological Sciences’ ethics board.

**Table 1:** Descriptive statistics: parent reported touchscreen use and developmental milestones in 6- to 36-month-olds .

	Age quartiles				Total	Total
	6–11 m	12–18 m	19–25 m	26–36 m	6–36 m	19–36 m
Age (months)						
*M*	8.99	14.40	21.94	30.64	**19.52**	**26.39**
*(SD)*	(1.82)	(2.19)	(2.07)	(3.07)	**(8.26)**	**(5.08)**
*N*	134	215	179	187	**715**	**366**
Own touchscreen (percentage)						
%	0	5.67	11.03	21.19	**9.62**	**16.22**
*N*	123	194	145	151	**613**	**296**
Touchscreen use (percentage)						
%	51.22	73.20	80.69	92.05	**75.20**	**86.49**
*N*	123	194	145	151	**613**	**296**
Touchscreen use (minutes)						
*M*	8.53	18.80	25.18	44.11	**24.45**	**34.81**
*(SD)*	(15.54)	(36.83)	(37.46)	(47.75)	**(38.98)**	**(43.96)**
*N*	123	194	145	150	**612**	**295**
First scroll (months)						
*M*	6.33	9.05	13.46	16.62	11.91	**15.11**
*(SD)*	(2.11)	(2.77)	(4.85)	(6.66)	(5.93)	**(6.06)**
*N*	65	152	119	130	466	**249**
First video (months)						
*M*	6.04	8.45	13.02	16.20	11.66	**14.67**
*(SD)*	(2.37)	(3.54)	(5.45)	(6.92)	(6.37)	**(6.45)**
*N*	58	126	116	126	426	**242**
Gross motor (months) Sitting						
*M*	5.64	5.71	5.49	5.86	5.68	**5.67**
*(SD)*	(0.98)	(1.25)	(1.13)	(1.52)	(1.25)	**(1.34)**
*N*	120	197	149	144	610	**293**
Walking						
*M*	10.56	12.10	12.81	12.91	12.58	**12.86**
*(SD)*	(0.53)	(1.75)	(2.15)	(2.52)	(2.20)	**(2.34)**
*N*	9	136	150	148	443	**298**
Fine motor (months) Pincer grip						
*M*	6.74	8.02	8.34	8.07	7.88	**8.21**
*(SD)*	(1.95)	(2.06)	(2.67)	(2.78)	(2.44)	**(2.72)**
*N*	96	185	131	121	533	**252**
Stack blocks						
*M*	9.50	12.10	13.26	13.33	12.91	**13.29**
*(SD)*	(1.52)	(2.37)	(3.70)	(4.65)	(3.81)	**(4.17)**
*N*	6	87	126	113	332	**239**
Language (months) First word						
*M*	7.97	10.72	12.24	11.21	11.11	**11.75**
*(SD)*	(2.09)	(2.55)	(3.30)	(3.52)	(3.24)	**(3.44)**
*N*	35	151	140	126	452	**266**
Two words						
*M*	8.00	13.98	17.20	16.62	16.38	**16.90**
*(SD)*	(2.83)	(2.62)	(3.79)	(4.49)	(4.15)	**(4.17)**
*N*	2	44	114	124	284	**238**

#### Demographic Information

Information was collected about the child’s age (mean age = 19.52 months, *SD* = 8.26 months) and sex (336 females), as well as mother’s educational level (a proxy for family SES; “What is the highest degree or level of education the mother of the child has completed?” Responses were “Not applicable,” *N* = 3; “School leaving qualification,” *N* = 20; “College,” *N* = 79; “University,” *N* = 294; and “Post-graduate,” *N* = 319).

#### Touchscreen Usage

Media questions were derived from existing questionnaires investigating touchscreen usage (Rideout, 2013; Linebarger et al., 2014; Ofcom, 2014). Parents were asked about (1) number of devices: ‘How many touchscreen devices do you have in your home?’ and ‘How many of these touchscreen devices belong to your child?; (2) frequency of child’s use: ‘On a typical day, how long does your child spend using a touchscreen device?’; and (3) age of first use: ‘How old was your child when he/she first did the following activities on a touchscreen device… Scrolled or touched the screen/Passively watched videos.’

#### Developmental Milestones

In order to assess the onset of key developmental milestones without having to complete an entire standardized assessment (e.g., Vineland Adaptive Behavior Scales, VABS-II; Sparrow et al., 2005), critical milestones from motor and language domains were chosen. All questions took the format: ‘At what age did he/she first…’ and in the current paper, we use data from one ‘early’ and one ‘late’ milestone, e.g., ‘Sit without support’ and ‘Walk independently’ (gross motor), ‘Pick up a small object with a pincer grip, i.e., with thumb and forefinger’ and ‘Stack at least three small blocks or other small objects; stack must not fall’ (fine motor), ‘Say their first word’ and ‘Put two or more words together’ (language).

### Statistical Analysis

Data were initially cleaned using scripts in SPSS (Ver. 22, IBM Corp., 2013) to remove any impossible values due to entry errors, e.g., more than 24 h per day on a touchscreen. In addition, one child’s reported daily touchscreen usage time was removed from the current analyses (a clear outlier of 1200 min per day which was >19 SD above the mean). Analysis was performed using SPSS and Stata 13 (StataCorp, 2013).

To assess the association between age of first touchscreen use and age of achieving developmental milestones, three separate partial correlations were run for gross motor skills (walking), fine motor skills (stacking blocks), and language (producing two-word utterances) with age of touchscreen use, covarying for the corresponding ‘early’ milestone (pincer grip, sitting and first word, respectively), mother’s education (a proxy for social economic status), age, and sex. Partial correlations were chosen as the retrospectively reported time of first touchscreen use and age of achieving milestones varied in the order of which occurred first, so the direction of effect cannot be inferred.

## Results

### How Does Touchscreen Use Change From 6 To 36 Months?

In our sample, only two of the 715 respondents had no touchscreen devices in their home, average ownership being 3.73 devices per household (*SD* = 1.50, range 0–14). Among 6- to 36-month-old infants and toddlers, 9.62% of children (59/613) had their own touchscreen. When split by age (quartiles 6–11, 12–18, 19–25, and 26–36 months) ownership increased from 0% among infants aged 6–11 months through to 21.19% for 26- to 36-month-olds (see **Table 1**).

**FIGURE 1:**
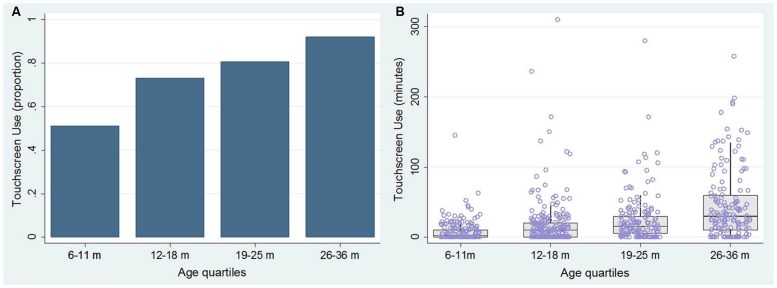
**(A)** The proportion of touchscreen users for each age quartile, from 6 and 36 months. **(B)** The relationship between age and mean daily touchscreen usage (minutes).

Overall, ~75% of our sample used a touchscreen, and analysis of touchscreen use by age showed that the proportion of users increased from 51.22% in 6–11 month-olds through to 92.05% by 25–36 months (see **Figure 1A**). However, within the children who did not use a touchscreen daily (i.e., ~25% of 6- to 36-month-olds) only 42.11% (64/152) reported no prior use of a touchscreen device at all. Within users, the average daily use between 6 and 36 months was 24.45 min (*SD* = 38.98, range: 0–310 min), which increased from 8.53 min per day at 6–11 months to 43.95 min at 26–36 months (see **Figure 1B**).

### Retrospectively Reported Touchscreen Use and Developmental Milestones

For all the following retrospectively reported data (i.e., age of first touchscreen use and achieving developmental milestones), our analyses include only toddlers 19 months and older (i.e., a median split; *N* = 366). This is to ensure that the majority of children have had the opportunity to achieve the developmental milestones and use a touchscreen device (see **Table 1**). In these toddlers, mean reported age of first touchscreen use was 13.13 months (*SD*: 6.05), with no significant difference between age of first scrolling (mean: 15.11 months, *SD*: 6.06) and video watching (14.67, *SD*: 6.45): *t*(225) = -1.17, *p* = 0.25.

For developmental milestones, a 2 (developmental level: early, late) ^∗^ 3 (domain: gross motor, fine motor, language) repeated measures ANOVA, showed a significant effect of domain [*F*(2,414) = 246.15, *p* < 0.001, $\eta_{p}^{2}$ = 0.54], with parents reporting earliest achievement for gross motor skills (estimated mean age = 9.15 months), followed by fine motor skills (estimated mean = 10.67 months) and then language (estimated mean = 14.06 months). There was also a significant effect of developmental level [*F*(1,207) = 1624.79, *p* < 0.001, $\eta_{p}^{2}$ = 0.89], with the ‘early’ milestones (sitting, pincer grip, and first word; estimated mean age = 8.38 months) achieved, as expected, before the ‘late’ milestones (walking, stacking blocks, and combining two words together; estimated mean age = 14.21 months). The developmental level by domain interaction was also significant [Greenhouse–Geisser corrected: *F*(1.81,374.92) = 22.39, *p* < 0.001, $\eta_{p}^{2}$ = 0.10], with less difference between the age of achieving the ‘late’ milestones compared to the ‘early’ milestones.

### How Does Touchscreen Usage Relate to Developmental Milestones in Toddlers?

**FIGURE 2:**
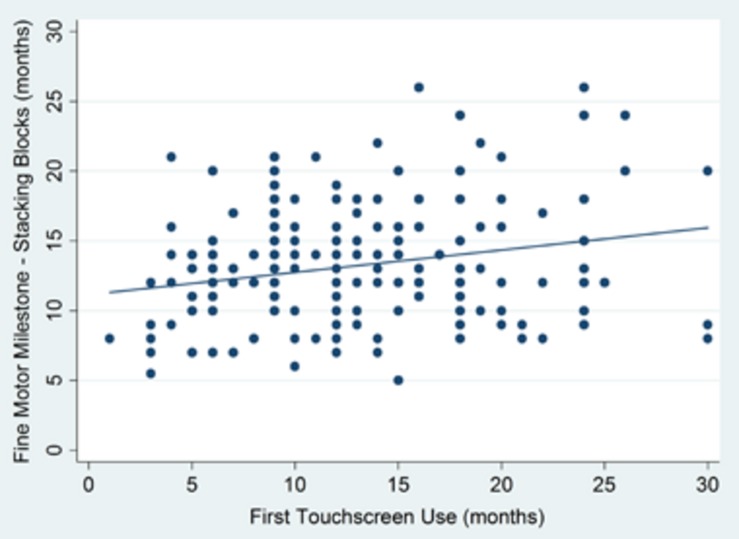
**Association between retrospectively reported age of first touchscreen use and achievement of fine motor milestone (stacking blocks)**.

Analyzing data from toddlers between 19 and 36 months of age, we tested whether age of first touchscreen use was correlated with age of achieving key developmental milestones: gross motor skills (walking), fine motor skills (stacking blocks), and language (producing two-word utterances), covarying for the corresponding ‘early’ milestone (pincer grip, sitting, and first word, respectively), mother’s education, age, and sex. No significant associations between age of first touchscreen use and either gross motor (walking: *r* = -0.08, *p* = 0.21) or language (combining two words: *r* = -0.02, *p* = 0.83) were found. However, age of first touchscreen usage was significantly associated with the fine motor milestone stacking blocks (*r* = 0.16, *p* = 0.03) (see **Figure 2**). To test whether type of usage was important, we ran separate partial correlations for age of first active scrolling of the screen and watching videos. Age of first scrolling was significantly associated with stacking (*r* = 0.16, *p* = 0.04), controlling for previous covariates (pincer grip, mother’s education, age, sex) and age of first watching videos. However, the association with age of first watching videos (controlling for scrolling) was not significant (*r* = 0.04, *p* = 0.62).

## Discussion

These results confirm the prevalence and rapid increase of touchscreen use over the first 3 years of life in a large UK-wide online sample. Contrary to the guidelines adopted by international parent-advisory agencies (Brown, 2011; Strasburger and Hogan, 2013) including the UK government (Public Health England, 2013), the majority (75.20%) of our 6- to 36-month-old sample of children had daily exposure to touchscreen devices, far exceeding the prescribed zero screen time for that age group. This figure is higher than reported in earlier studies examining touchscreen use up to 24 months of age (Rideout, 2013; Cristia and Seidl, 2015) but similar to the exposure reported in a recent sample from Northern Ireland (Ahearne et al., 2015) and to a low SES American sample (Kabali et al., 2015). Even within the 25% who did not report daily touchscreen use, only 42.1% reported that their child had never used a touchscreen. These results indicate that within our sample, touchscreen devices are a common part of a toddler’s media environment and everyday sensory/cognitive stimulation.

The representativeness of our sample to the UK population is not known, as the recruitment was not random and participants may have volunteered due to a pre-existing interest in the topic of media and child development. The proportion of high SES families was also overrepresented in our sample with 86% with degree level education or above. However, a recent UK government survey confirms that the majority of families with somewhat older children (3- to 4-year-olds) own at least one tablet computer (65%) and use it regularly (39%; Ofcom, 2014). Our higher touchscreen exposure percentage (75.20% versus Ofcom’s 39%) may be due to the fact that Ofcom does not include touchscreen smartphones within results for tablets (smartphone availability is measured separately; e.g., 41% of 5- to 15-year-olds own a smartphone; Ofcom, 2014). This is supported by a survey of 3- to 5-year-old children conducted by The National Literacy Trust which reported 72.9% access to a touchscreen device in the home including smartphones (Formby, 2014). Thus, while it does not appear that our sample is made up of families with uncharacteristically high media exposure, it is possible that there are more subtle differences in the way in which devices are being used by higher SES families. Future studies should attempt to gather a randomly selected, representative sample, or target families with low-media use to ensure representation of the full range of media environments.

One objective of this study was to address popular fears that early exposure to touchscreen devices may negatively impact toddler development (Carr, 2010; Sigman, 2012; Greenfield, 2015). In order to gather data on early development in a large online sample without the need for a long standardized questionnaire (e.g., Vineland Adaptive Behavior Scales) or observational assessment (e.g., Mullen; Mullen, 1995), we chose the age of achieving key developmental milestones: fine motor (i.e., stacking blocks), gross motor (i.e., walking), language (i.e., saying two-word utterances). However, this approach yields only single-item measures of developmental milestones. In reality of course, abilities such as walking actually develop over a period of time, from a first shaky step to confident locomotion. Future studies should collect more detailed developmental measures longitudinally, at the time they are emerging. An additional limitation is the retrospective reporting of milestones, which are subject to recall bias (Sudman and Bradburn, 1973), as well as the fact that we do not know how reliable parents are in remembering aspects of first touchscreen use. Our results did not show any evidence for negative associations between touchscreen use and developmental milestones, but there was a significant positive association between the retrospectively reported age of achieving the fine motor milestone and the age at which the child first used a touchscreen. Specifically, this relationship was only present for the child’s age of first actively controlling the screen by scrolling or touching, and not for watching videos.

The average age of reaching the fine motor milestone – stacking three or more blocks – was 13.29 months (*SD* = 4.17), which is consistent with the 12–15 months age window expected for the development of block stacking (Gerber et al., 2010). The positive association suggests that infants who are actively using a touchscreen earlier are also developing earlier fine motor abilities observable with real objects. However, given that both the milestone and the age of first use are retrospectively reported and may occur in either order we cannot currently interpret the direction of this effect. It may be that infants with developmentally advanced fine motor skills are more likely to actively scroll, touch and control a touchscreen device when given the opportunity, in the same way that they will apply their newfound skills to any object placed before them. Alternately, exposure to a highly stimulating, rewarding and responsive touchscreen device prior to the onset of their advanced fine motor skills may encourage experimentation of finger and hand control which ultimately transfers to real-world objects. In order to know which of these hypothesized mechanisms is causing the effect, future studies will need to chart both touchscreen use and motor development longitudinally using more fine-grained and precise measurement.

Evidence for a relationship between active screen-based media use and fine motor skill has previously been reported in older children and adults (for review, see Green and Bavelier, 2008). Touchscreen phone use is related to increased fingertip somatosensation and associated brain activity in adults (Gindrat et al., 2015), manual dexterity and visual motor skills can be trained by videogames including specialist skills such as laparoscopic surgery (Lynch et al., 2010) and piloting an aircraft (Gopher et al., 1994). Transfer of the manual skill trained by screen-based media to the real world is often dependent on specificity of the trained virtual skill and its similarity with the real skills (Green and Bavelier, 2008). For example, a study assessing the relationship between general computer use and development in 38- to 61-month-olds found no relationship with visuomotor or gross motor development even though advantages in school readiness and cognitive development were found (Li and Atkins, 2004). To assess causality in the direction of the relationship between active touchscreen use and real world fine motor development, future studies should utilize an intervention design in which specific manual gestures/skills are encouraged in infants using a touchscreen early in development.

In terms of the assessed gross motor (walking) and language milestones (two word utterances) our analyses did not reveal any relationship – positive or negative – with the age of first using a touchscreen. The average reported age of walking onset in our toddler sample (12.86 months, *SD* = 2.34) is consistent with the expected mean walking onset (12 months; Onis, 2006; Gerber et al., 2010) as is the age of first using two-word utterances (our sample: 16.90 months, *SD* = 4.17; compared to mean onset 17–18 months: Fenson et al., 2000; Gerber et al., 2010). Our results can neither confirm nor deny the *displacement hypothesis* (Strasburger et al., 2012), which states that the time a child spends engaged with a screen limits the time they have to do other activities, leading to reduced physical activity (Sisson et al., 2010; although see Taveras et al., 2007) or face-to-face communication (Huttenlocher, 1998; Hart and Risley, 2003; Rowe, 2012; Sosa, 2016). Although, we do not have a measure of physical activity or social interaction in our sample, we do not find any evidence that touchscreen usage is displacing other forms of physical exploration that relate to the onset of walking or the social and linguistic stimulation that facilitates spoken language when other factors such as SES, sex, and age are controlled for. This absence of displacement may be, in part, because touchscreen devices are inherently portable (thus facilitating mobility), can be used collaboratively by multiple people (for an overview of pre-schooler/parent tablet co-use see Marsh et al., 2015) and are increasingly being used in parallel with other media such as TV (74% of 14- to 17-year-olds report using a smartphone whilst watching TV; Accenture, 2015). In addition, it may be that early touchscreen use impacts language and gross motor only later in development, when these skills are more advanced (e.g., vocabulary size, physical activity), rather than the early milestones assessed here.

Our findings are the first attempt to identify the impact of the recent introduction of touchscreen devices into the early environment of toddlers on development. The results of the current study provide no evidence of a negative association between toddlers’ use of touchscreen devices and developmental outcomes and even suggest a positive association with fine motor development. However, our current analysis is limited by the fact that we do not have a measure of how much each child was using a touchscreen prior to reaching developmental milestones. Our only measure of the amount of usage is reported at the child’s current age and we do not know how this relates to earlier usage. To examine whether the association between early touchscreen use and age of reaching developmental milestones is “dosage” dependent or varies with other usage factors (such as co-use, physical context, or type of use), future studies will need to use more reliable methods of tracking current touchscreen use such as media diaries or objective measures (e.g., device monitoring). Also, our present analysis does not report other aspects of development that may also be associated with early touchscreen use such as eyesight problems (e.g., increased myopia in children who read intensely; Ip et al., 2008), muscular and skeletal pain and problems due to excessive use (e.g., phone use in adults; Berolo et al., 2011), sleep problems (e.g., videogame use in children; Dworak et al., 2007), emotion and conduct problems (e.g., childhood TV predicting adult problems; Robertson et al., 2013), or cognitive development such as attention control and executive function (e.g., the immediate impact of fast-paced TV viewing; Lillard and Peterson, 2011). More precise lab-based assessments of these factors are required along with the detailed analysis of touchscreen usage. Future intervention studies that can control for pre-existing differences in these groups and help to address questions of causality will also be important, although the ethical issues of how to introduce a potentially negative influence into a child’s environment must be addressed first (Bavelier et al., 2010). A more nuanced charting of the exact ways in which children are using the touchscreen, as well as the pedagogical and age-appropriateness of the apps (Hirsh-Pasek et al., 2015), is required. Given the inherent flexibility of these devices, not all use can be considered to have the same impact on development (Christakis, 2014). This view is supported by the differential association we find between the age of fine motor milestone achievement and touchscreen scrolling versus video watching.

Our results suggest that touchscreen use by toddlers is prevalent in the UK and only likely to increase given the increasing computational intelligence and adoption of touch as a mode of interaction by almost all household appliances, toys and even clothing (e.g., smartwatches and running gear). The intuitive nature of touchscreens for pre-linguistic children (Cristia and Seidl, 2015) means that the current recommendations for zero screen time for children under 2 years is out of line with the reality of the current home media environment of most toddlers and difficult to enforce by parents who themselves are conducting more of their lives through such devices. Parents may also be considering touchscreen devices as exempt from the “no screens” guidelines, as has previously been reported for infant and toddler use of videochat (McClure et al., 2015). Our evidence that children who scroll touchscreen devices earlier may develop fine motor control earlier is the first indication of how our current generation are adapting to their new media environment and setting the foundation for a life spent interacting with such devices. How such exposure relates to long-term development, educational achievement and impacts future society are pressing research questions facing developmental science.

## Author Contributions

RB: conceptualized and designed the study, analyzed the data, and wrote the paper; ISU: designed and implemented the questionnaire, processed the data, and contributed to drafts of the paper; CC: coordinated questionnaire recruitment, processed the data, and contributed to drafts of the paper; AK-S: conceptualized and designed the study and contributed to drafts of the paper; TS: conceptualized and designed the study, analyzed the data and wrote the paper.

## Conflict of Interest Statement

The authors declare that the research was conducted in the absence of any commercial or financial relationships that could be construed as a potential conflict of interest.
